# Role of Gut Microbiota and Metabolomics in Predicting Response to Vedolizumab in Inflammatory Bowel Disease: A Systematic Review

**DOI:** 10.3390/pharmaceutics17040476

**Published:** 2025-04-06

**Authors:** Vaidota Malinauskiene, Elena Cijauskaite, Goda Sadauskaite, Ieva Stundiene

**Affiliations:** Clinic of Gastroenterology, Nephrourology and Surgery, Institute of Clinical Medicine, Faculty of Medicine, Vilnius University, 01513 Vilnius, Lithuania; elena.cijauskaite@santa.lt (E.C.); goda.sadauskaite@santa.lt (G.S.); ieva.stundiene@santa.lt (I.S.)

**Keywords:** inflammatory bowel disease, Crohn’s disease, ulcerative colitis, gut microbiota, short-chain fatty acids, vedolizumab

## Abstract

**Background**: This review explores the impact of gut microbiota profiles in predicting the response to anti-integrin biologic therapy, particularly vedolizumab, in inflammatory bowel disease (IBD) patients. IBD, encompassing Crohn’s disease and ulcerative colitis, is a chronic inflammatory condition with a growing prevalence linked to industrialization and lifestyle changes. Disruption in the gut microbiota balance, characterized by reduced diversity and altered short-chain fatty acid (SCFA) production, is associated with IBD and its symptoms. Current pharmacological treatments target healing and remission, with vedolizumab offering a gut-selective treatment approach. **Methods**: A search of the literature was performed on the relationship between anti-integrin treatment and the microbiome profile in IBD. Articles were examined from the PubMed, Medline, Cochrane, and Web of Science databases. **Results**: This review identified five human studies investigating the relationship between gut microbiome composition, SCFAs, and response to vedolizumab, revealing an increased abundance of beneficial bacteria and levels of SCFAs like butyrate in remission cases. Despite promising findings, the small sample sizes and limited scope of the existing studies highlight the need for larger, comprehensive research. **Conclusions**: This review underscores the potential of gut microbiome and metabolite profiling as non-invasive biomarkers for IBD severity and treatment outcomes, advocating for personalized therapeutic strategies to enhance efficacy. The insights gained could lead to novel diagnostic and treatment modalities, although further validation is necessary to fully understand the intricate connections between gut microbiota and IBD prognosis.

## 1. Introduction

Inflammatory bowel disease (IBD) represents a diverse group of chronic inflammatory conditions that encompass the two specific subtypes of Crohn’s disease (CD) and ulcerative colitis (UC), affecting approximately 6.8 million people worldwide [1,2]. The incidence and prevalence of IBD have been rising in tandem with the industrialization, lifestyle changes, and urbanization of modern societies [1]. IBD is an idiopathic inflammatory gastrointestinal disease that can result from various etiologies, including immune system responses, genetic predisposition, and alterations in the gut microbiota [3,4].

The gut microbiome is a complex ecosystem comprising a wide variety of microorganisms (viruses, bacteria, fungi, and archaea) that predominantly reside in the large intestine [5]. Six dominant phyla primarily constitute a healthy human gut microbiota, including Euryarchaeota, Firmicutes, Actinobacteria, Proteobacteria, Verrucomicrobia, and Bacteroidetes [6,7]. Although the gut microbiota is generally stable throughout a person’s lifetime, factors such as dietary modifications, environmental changes, pathogenic infections, and lifestyle choices can disrupt this balance [8]. The gut microbiome in IBD patients is characterized by reduced alpha diversity and lower levels of beneficial bacteria or fungi, such as *Bifidobacteria*, along with an increase in pathogenic bacteria like Proteobacteria, *Fusobacterium* species, and *Ruminococcus gnavus*, as well as higher levels of *Candida albicans* and lower levels of *Saccharomyces cerevisiae* compared to healthy individuals [9,10]. Significant alterations in gut microbial metabolites, particularly short-chain fatty acids (SCFAs) such as acetate, propionate, and butyrate, have been observed. These SCFAs are crucial for maintaining intestinal homeostasis, promoting epithelial barrier function, serving as the primary energy source for colonocytes, modulating histone deacetylase activity, and exerting anti-inflammatory effects in the intestinal mucosa [11,12,13]. According to studies, patients with active IBD had lower fecal amounts of SCFAs [14,15].

The primary objective of current pharmacological treatments for IBD is transmural healing in Crohn’s disease and histological healing in ulcerative colitis [16]. Presently, the medications used in IBD treatment include corticosteroids, aminosalicylates, monoclonal antibodies targeting integrins, anti-TNF agents, IL-12/23 inhibitors, and Janus kinase inhibitors (JAKs) [8]. The current treatments for IBD, including immunosuppressants and biologics, are limited by variable efficacy, high recurrence rates, and significant side effects. Furthermore, some patients experience primary nonresponse or develop resistance over time [1,17]. Given the involvement of the gut microbiota in the pathogenesis of IBD, researchers have recently begun to investigate how these therapies impact the gut microbiome.

As an IgG1 humanized monoclonal antibody that binds to α4β7 integrins, vedolizumab is a biologic agent that exclusively targets the gastrointestinal tract [18,19]. Some recent studies have looked into how the gut microbiome and metabolomic profiles may relate to predicting patient responses to vedolizumab [17,20,21,22,23]. This systematic review presents the latest findings on how gut microbiome characteristics could predict the efficacy of anti-integrin biologic treatments in patients with inflammatory bowel disease (IBD).

## 2. Methods

### 2.1. Search Strategy

A search of the digital literature was performed on the relationship between anti-integrin treatment and the microbiome profile in inflammatory bowel disease (IBD). As of June 1, 2024, articles were examined from the PubMed, Medline, Cochrane, and Web of Science databases. The following search phrases were used: “anti-integrin therapy AND microbiome”, “vedolizumab AND microbiome”, “anti-integrin therapy AND microbiota”, and “vedolizumab AND microbiota”. No time restrictions were used for publications. Two separate reviewers assessed the titles and abstracts of the collected articles to pinpoint potentially relevant studies. Discussions or, if required, consultation with a third reviewer were used to settle disagreements. For studies that satisfied the inclusion requirements, full-text publications were acquired. References of selected articles were manually searched for additional eligible studies to ensure a search.

This study has been registered with PROSPERO (the International prospective register of systematic reviews) under the registration number CRD420251021185.

### 2.2. Eligibility Criteria

Inclusion criteria comprised clinical studies that investigated the impact of anti-integrin therapies on gut microbiota in IBD patients, with a focus on interventions involving vedolizumab. Studies were required to report microbiome composition changes and clinical outcomes. Exclusion criteria included non-clinical trials, non-peer-reviewed articles, abstracts only, studies not reporting relevant microbiome data, and animal module studies.

Data were extracted independently by two reviewers using a predetermined data extraction form. Extracted information included study design, participant characteristics, intervention details, outcomes related to microbiome changes, and key findings.

This review of the available data on the impact of vedolizumab on gut microbiota in patients with IBD was prepared and updated in accordance with the PRISMA 2020 Checklist (Table S1) [24].

## 3. Results

**Figure 1:**
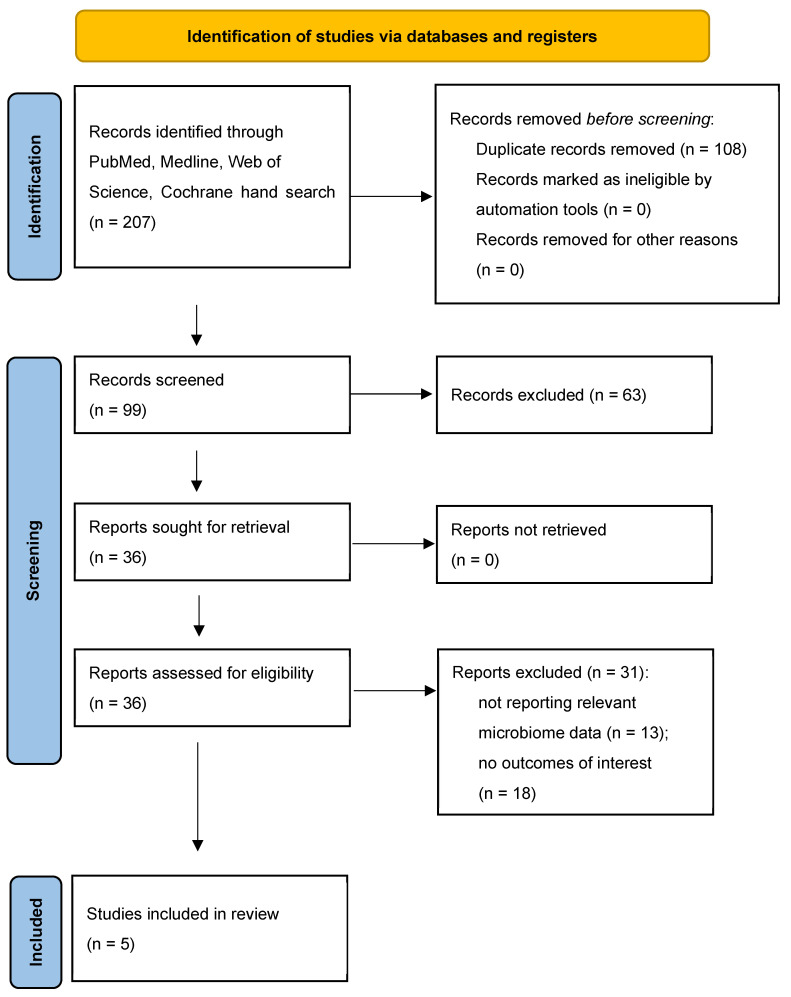
Final selection of articles.

Following a review of the current literature, out of 207 articles, five studies that met the predefined eligibility criteria and were incorporated into the final analysis [17,20,22,23,25]. The process of identification and screening is described in Figure 1 and literature search workflow is in Figure S1.

The characteristics of the included studies and patients are presented in Table 1. The included study findings are summarized in Table 2.

## 4. Discussion

### 4.1. Gut Microbiome Composition Shifts

Dysbiosis, characterized by a reduction in microbial diversity and an increase in pathogenic bacteria, is a hallmark of IBD and plays a critical role in its pathogenesis [4,26]. Patients with IBD exhibit significant alterations in their gut microbiota, including an overgrowth of harmful bacteria such as *Bacteroides fragilis* and a reduction in beneficial species like *Eubacterium rectale*, *Bifidobacterium longum*, *Faecalibacterium prausnitzii*, and *Roseburia intestinalis*, compared to healthy individuals [27,28,29]. Therapeutic interventions, such as anti-TNF agents and vedolizumab, may partially restore microbial balance, and emerging evidence suggests that microbiome composition and diversity can influence treatment responses [4,21]. This highlights the rationale for targeting the microbiome in IBD therapy to complement existing approaches and improve patient outcomes.

The most extensively studied drug class for its effects on the gut microbiome is anti-TNF therapy [30]. According to a study by Aden et al. [21], anti-TNF therapy in IBD restored fecal microbiome diversity to levels comparable to those of the control group. Additionally, another study demonstrated that in patients treated with infliximab, the number of harmful bacteria was reduced, and the diversity and richness of the fecal microbiota in CD patients increased significantly [31]. Moreover, in a study conducted by Caenepeel et al. [25], patients with dysbiotic Bacteroides2 enterotype at baseline experienced significantly higher remission rates with anti-TNF therapy compared to those treated with vedolizumab.

In a study by Liu et al. [17], the fecal microbiome of UC patients receiving vedolizumab was compared to that of healthy controls. At the phylum level, the remission group showed a considerably higher enrichment of *Verrucomicrobiota* at week 14 than the non-remission group. Additionally, *Ruminococcus* and *Akkermansia* were substantially more prevalent at the genus level in remission patients than in non-remission patients. *Akkermansia muciniphila*, a mucus-degrader within the *Verrucomicrobiota* phylum, has been shown to colonize the gut and can both trigger the production of homeostatic IgG and inhibit the growth of pathogenic bacteria [32].

Another study analyzed by Ananthakrishnan et al. [20] revealed that among CD patients who achieved remission at week 14, alpha-diversity was significantly higher, and *Roseburia inulinivorans* and a *Burkholderiales* species were more common at baseline. Additionally, in the same study, a more diverse microbial composition at baseline was associated with predicting clinical remission by week 14. Furthermore, early microbial changes persisted for up to one year in responders. The development of IBD has consistently been associated with a less diverse microbiome, and factors such as stress, diet, and medications that decrease gut diversity raise the risk of IBD [33,34]. A less damaged mucosal barrier and a more diverse microbiome at baseline may lead to reduced colonic inflammation and a better response to treatment, possibly due to the anti-inflammatory effects of prominent bacteria and metabolites. This can occur because prominent bacteria and metabolites may have an anti-inflammatory effect. In a study by Colman et al. [23], the change in alpha diversity between week 0 and week 2, along with baseline beta diversity, was associated with corticosteroid-free remission at week 14.

A study by Lee et al. [22] revealed higher abundances of *Bacteroides* species (*B. ovatus*, *B. stercoris*) and *Bifidobacterium longum* among patients starting vedolizumab and achieving remission at week 14. Some of the bacterial strains linked to clinical remission have also shown efficacy as independent microbiome-directed therapies. *B. ovatus* monotherapy outperformed fecal transplantation in achieving clinical remission in a mouse colitis mode [35]. Moreover, some studies have shown that specific strains of *B. ovatus* can suppress inflammation in the bowel [35,36].

Most studies have demonstrated that therapeutic interventions, including vedolizumab, can restore gut microbiome diversity and promote remission. Studies have shown that positive treatment outcomes are associated with an increased abundance of beneficial bacteria and microbial diversity at baseline. These findings suggest that analyzing gut microbiota could be crucial for predicting treatment responses and personalizing IBD management. Further exploration of microbiome-directed therapies offers potential for enhancing inflammation control and remission in IBD patients.

### 4.2. Short-Chain Fatty Acids and Other Metabolites Changes

Of special significance are bacterial species that break down indigestible dietary fibers and produce substances that are beneficial to the intestinal mucosa. Short-chain fatty acids (SCFAs), such as acetate, propionate, and butyrate are very important metabolites for maintaining intestinal balance. For instance, butyrate not only serves as the primary energy source for colonocytes but also acts as an anti-inflammatory agent that helps to preserve intestinal balance [37,38]. The majority of butyrate-producing bacteria in the human gut belong to the phylum Firmicutes, including *Eubacterium rectale*, *Roseburia* spp., *Faecalibacterium prausnitzii*, and *Clostridium leptum* [39]. The evidence suggests that both the intestinal mucosa and feces of IBD patients have reduced concentrations of dominant SCFA-producing bacteria, as well as steady-state levels of SCFAs in these tissues also being lower when compared to healthy controls [28,29,40]. A study by Takahashi et al. [28] found that patients with Crohn’s disease had significantly fewer butyrate-producing bacterial species, including *Blautia faecis*, *Roseburia inulinivorans*, *Ruminococcus torques*, *Clostridium lavalense*, *Bacteroides uniformis*, and *Faecalibacterium prausnitzii*, compared to healthy controls.

In animal studies, butyrate treatment improved colonic lesions in rats with colitis and reduced levels of IL-17 in the colonic mucosa and plasma. Butyrate therapy also reduced colitis scores in both acute and chronic colitis [41,42]. Furthermore, individuals with week 14 remission had considerably higher serum bile acid levels, particularly secondary bile acids. Human studies of fecal microbial transplantation in IBD patients have shown that the presence of *Eubacterium* and *Roseburia* species, along with SCFA production, is associated with remission in IBD [43]. In our analyzed studies, the baseline butyric acid and isobutyric acid levels as well as levels of SCFAs producing beneficial bacteria were significantly higher in the remission groups compared to the non-remission groups [17,20]. The higher concentrations of SFCAs and SCFA-producing bacteria, such as *Verrucomicrobiota*, *B. ovatus*, *R. inulinivorans*, were linked to successfully achieved remission at week 14 of anti-integrin therapy [20,22]. In the pediatric population, a greater abundance of butyrate-producing bacteria was linked to an early response to vedolizumab, suggesting that microbial analysis could be helpful in conjunction with biological selection [23]. Studies focusing on anti-TNF therapy have also shown that responders to anti-TNF medication had more abundant SCFA-producing bacteria than non-responders [44,45].

In conclusion, the presence and activity of butyrate-producing bacteria in the gut microbiome are crucial for maintaining intestinal health and may significantly influence disease outcomes in IBD patients. The production of SCFAs, particularly butyrate, plays a dual role in providing essential energy sources for colonocytes and exerting potent anti-inflammatory effects, thus supporting intestinal homeostasis [46]. The observed deficiency of these bacterial species in IBD patients highlights a potential target for therapeutic intervention. Studies, including those focused on butyrate supplementation and microbial transplantation, underline the therapeutic potential of enhancing SCFA-producing bacterial populations to achieve and maintain remission. The correlation between higher baseline levels of beneficial bacteria and favorable responses to therapies such as vedolizumab emphasizes the importance of integrating microbiome composition analysis into clinical decision-making. This approach not only aids in predicting treatment responses but also paves the way for personalized IBD management strategies that harness the gut microbiota’s capabilities, thereby optimizing patient outcomes. Further research is warranted to explore this promising avenue and its broader application in therapeutic settings.

## 5. Limits of the Current Knowledge and Future Research Perspectives

There are certain limitations to the knowledge provided by the existing studies. Firstly, most studies included relatively small sample sizes of remission and non-remission patients, and all study cohorts were based in a single center. Secondly, in most studies, remission was assessed based on clinical indicators at week 14 rather than biochemical, fecal, or endoscopic outcomes. A study by Lee et al. [22] included endoscopic results, but these were available for less than half of the group. In the same study, statistical power was diminished because some patients did not provide baseline serum and stool samples. A study conducted by Caenepeel et al. [25] tackled some of these limitations by examining a larger cohort of microbiome profiles involving 296 patients, including 123 who were on vedolizumab. The researchers evaluated therapeutic response using endoscopy, patient-reported outcomes, and fecal calprotectin levels.

Furthermore, most cohorts consisted predominantly of refractory patients, many of whom had previously received anti-TNFs. None of the studies mentioned additional medications that patients were taking while on vedolizumab, such as 5-ASA (mesalazine) or azathioprine. These medications may also impact gut microbiota diversity and induce changes in the microbiota of IBD patients both before and during therapy [47]. Moreover, studies could clarify how gut microbial patterns and dietary influences interplay with treatment efficacy [48].

Recent studies have linked biologic therapy—mainly anti-TNFs—with the restoration of the gut microbiome in IBD and other autoimmune diseases [49,50,51]. Several noteworthy bacterial species modulated by the treatment have been identified. These findings suggest that gut microbiota profiling could lead to biomarkers for personalized treatment strategies, disease activity assessment, and anti-TNF treatment response prediction. Gut microbiome alterations and metabolite changes were also observed in our analyzed studies of patients receiving vedolizumab. To classify IBD patients and provide personalized treatment for optimal outcomes, the gut microbiota can be investigated as a potential strategy. It offers promising biomarkers for the non-invasive evaluation of IBD severity and vedolizumab treatment efficacy. Although recent findings have linked the recovery and changes in gut microbiota with biologics, there is a pressing need for larger, multi-center, longitudinal studies, and multi-omics approaches that account for these variables and include both clinical and biochemical markers of remission. Ultimately, this knowledge may pave the way for the development of personalized diagnostic and therapeutic strategies, enhancing management and improving outcomes for patients with IBD.

## 6. Conclusions

The function of the gut microbiota and its potential impact on the therapeutic response to vedolizumab, a gut-selective anti-integrin biologic, in patients with IBD are the main topics of this review. Current evidence signifies that changes in gut microbiome composition and metabolite profiles, particularly the abundance of short-chain fatty acid (SCFA)-producing bacteria, are related with IBD remission. Higher levels of SCFAs also contribute to intestinal homeostasis and reduce inflammation, establishing them as potential biomarkers for treatment response. Nonetheless, the reliability and applicability of the reviewed studies are constrained by their small sample sizes, single-center cohorts, and failure to consider other factors, such as diet. These limitations highlight the need for larger, longitudinal, multi-center studies. These should incorporate diverse patient populations and detailed evaluations of diet and concurrent medications. A better understanding of how the microbiota and IBD interact can lead to more personalized treatment strategies, optimizing therapeutic efficacy and better patient outcomes. Further research in this field could pave the way for non-invasive diagnostic and prognostic tools, transforming the management of IBD.

## Figures and Tables

**Table 1:** Included studies’ characteristics.

Studies	Country	Study Design	Patients			Biologic Therapy Type		
			UC, n	CD, n	Controls, n	Anti-TNF, n	Vedolizumab, n	Ustekinumab, n
Liu et al. [17], 2023	China	Prospective cohort study	42	0	11	0	29	0
Ananthakrishnan et al. [20], 2017	USA	Prospective cohort study	43	42	0	0	85	0
Lee et al. [22], 2021	USA	Prospective cohort study	77	108	0	79	85	21
Colman et al. [23], 2022	USA	Prospective observational study	22	52	0	0	74	0
Caenepeel et al. [25], 2024	Belgium	Prospective cohort study	93	203	0	140	123	65

UC: ulcerative colitis, CD: Crohn’s disease; anti-TNF: tumor necrosis factor inhibitors; USA: United States of America.

**Table 2:** Clinical studies investigating the impact of anti-integrins on gut microbiome in inflammatory bowel disease patients.

Reference	Participants	Main Findings of the Study	Other Metabolites Investigated
**Liu et al.** [17]	13 inactive to mild UC patients. 29 moderate to severe UC patients. 11 healthy controls.	The remission group exhibited a significantly higher abundance of *Verrucomicrobiota* at the phylum level compared to the non-remission group. At baseline, there was a notably greater diversity of *Verrucomicrobiota* at the phylum level in the remission group compared to the non-remission group.	In the remission group, the concentrations of butyric acid and isobutyric acid were higher compared to the non-remission group at baseline. *Verrucomicrobiota*, butyric acid, and isobutyric acid combinationenhanced the ability to diagnose early remission to anti-integrin therapy.
**Ananthakrishnan et al.** [20]	43 UC patients. 42 CD patients. Most had previously failed an anti-TNF agent.	Alpha diversity was significantly higher among patients achieving remission at week 14. *Roseburia inulinivorans* and *Burkholderiales* species were more abundant at baseline among CD patients achieving week 14 remission.	Branched-chain amino acid synthesis was more abundant in baseline samples from CD patients who achieved remission. Taxonomic profiles at the level of genus, family, or class were less effective than information at the species level. Early microbial changes lasted for up to 1 year in responders.
**Lee et al.** [22]	The study cohort (n = 185; 108 CD, 77 UC): 79 anti-TNF. 21 ustekinumab. 85 vedolizumab.	Among patients initiating anti-integrin therapy, remitters had increased abundances of *Bifidobacterium longum* and *Bacteroides species* (*B. ovatus*, *B. stercoris*).	Patients with week 14 remission had higher abundance of serum bile acids, especially secondary bile acids in baseline serum samples. CASP8 was significantly associated with vedolizumab and inversely associated with clinical remission with anti-integrin therapy.
**Colman et al.** [23]	74 vedolizumab patients with IBD 52 CD 22 UC Children and young adults	The change in alpha diversity between weeks 0 and 2 and the baseline beta diversity (measured with shotgun metagenomic sequencing) predicted week-14 vedolizumab trough levels. The latter was linked to steroid-free remission by week 14.	Pre-infusion vedolizumab concentration values of 37 μg/mL and 20 μg/mL were the most accurate indicators of steroid-free clinical remission at infusion.
**Caenepeel et al.** [25]	The study cohort (n = 296; 203 CD, 93 UC): 140 anti-TNF. 65 ustekinumab. 123 vedolizumab.	Remission rates for patients hosting dysbiotic *Bacteroides2* enterotype at baseline were significantly higher with anti-TNF than with vedolizumab. A significant change in gut microbiota composition was associated with anti-TNF therapy but not with vedolizumab.	Positive *Bacteroides2* carrier status and higher fecal calprotectin levels both significantly decreased the chance of achieving remission after vedolizumab therapy.

UC: ulcerative colitis, CD: Crohn’s disease; IBD: inflammatory bowel disease; anti-TNF: tumor necrosis factor inhibitors.

## Data Availability

No new data were created or analyzed in this study. Data sharing is not applicable to this article.

## Supplementary Materials

The following supporting information can be downloaded at: https://www.mdpi.com/article/10.3390/pharmaceutics17040476/s1, Table S1: PRISMA 2020 Checklist; Figure S1: Literature search workflow.
